# Real World Feedback: How Well Did the Virtual Training Academy Prepare California's COVID-19 Contact Tracing Workforce?

**DOI:** 10.3389/fpubh.2022.857674

**Published:** 2022-06-28

**Authors:** Miranda Westfall, Maeve Forster, Olivia Golston, Kelly D. Taylor, Karen White, Michael J. A. Reid, Alina Dorian, Michael L. Prelip, Shira Shafir

**Affiliations:** ^1^Fielding School of Public Health, University of California, Los Angeles, Los Angeles, CA, United States; ^2^Institute for Global Health Sciences, University of California, San Francisco, San Francisco, CA, United States

**Keywords:** COVID-19, case investigation, contact tracing, public health workforce, public health preparedness

## Abstract

To effectively respond to the COVID-19 pandemic, California had to quickly mobilize a substantial number of case investigators (CIs) and contact tracers (CTs). This workforce was comprised primarily of redirected civil servants with diverse educational and professional backgrounds. The purpose of this evaluation was to understand whether the weeklong, remote course developed to train California's CI/CT workforce (i.e., Virtual Training Academy) adequately prepared trainees for deployment. From May 2020 to February 2021, 8,141 individuals completed the training. A survey administered ~3 weeks post-course assessed two measures of overall preparedness: self-perceived interviewing proficiency and self-perceived job preparedness. Bivariate analyses were used to examine differences in preparedness scores by education level, career background, and whether trainees volunteered to join the COVID-19 workforce or were assigned by their employers. There were no significant differences in preparedness by education level. Compared to trainees from non-public health backgrounds, those from public health fields had higher self-perceived interviewing proficiency (25.1 vs. 23.3, *p* < 0.001) and job preparedness (25.7 vs. 24.0, *p* < 0.01). Compared to those who were assigned, those who volunteered to join the workforce had lower self-perceived job preparedness (23.8 vs. 24.9, *p* = 0.02). While there were some statistically significant differences by trainee characteristics, the practical significance was small (<2-point differences on 30-point composite scores), and it was notable that there were no differences by education level. Overall, this evaluation suggests that individuals without bachelor's degrees or health backgrounds can be rapidly trained and deployed to provide critical disease investigation capacity during public health emergencies.

## Introduction

Case investigation and contact tracing are core public health strategies used to interrupt transmission of SARS-CoV-2, the virus that causes the coronavirus disease 2019 (COVID-19) Case investigation is the identification, investigation, and support of individuals with confirmed and probable diagnoses of COVID-19, and contact tracing is the subsequent identification, monitoring, and support of their close contacts who may have been exposed to the virus (1). Comprehensive and timely case investigation and contact tracing can reduce morbidity and mortality by providing education and resources for cases and contacts to adhere to isolation and quarantine guidance and therefore break the chain of infection (2, 3). While public health departments in the United States (U.S.) maintain contact tracing programs for ongoing prevention and control of transmissible diseases such as tuberculosis, the size of this workforce was quickly deemed insufficient to respond to the COVID-19 pandemic (4, 5). The National Association of County and City Health Officials estimated that the national contact tracing workforce capacity would need to double, from approximately 15 professionals to 30 professionals per 100,000 population in order to adequately contain COVID-19 (5). In California (CA), the contact tracing workforce gap was estimated to be ~10,000 professionals.

To bridge this workforce gap, public health experts at the California Department of Public Health (CDPH), the University of California, San Francisco (UCSF) and the University of California, Los Angeles (UCLA) were mobilized to develop and scale a virtual training program to rapidly train a large, competent workforce of case investigators (CIs) and contact tracers (CTs). This workforce was primarily redirected civil servants from county or state departments. Thus, trainees had diverse educational and professional backgrounds, and many had no public health knowledge or experience. In addition, while some staff volunteered to support the COVID-19 response, some were mandated to do so in their obligated role as Disaster Service Workers during State emergencies (6). As a result, trainees may have had varying levels of willingness to work as CIs/CTs. The purpose of this evaluation is to determine whether the program was effective in training a contact tracing workforce diverse in professional backgrounds and motivations to join the workforce.

## Methods

### Program Description

The COVID-19 Virtual Training Academy (VTA) for Case Investigators & Contact Tracers is a weeklong, remote learning course that combines self-study modules, live didactic webinars, and interactive skill development labs through which trainees build knowledge and practice skills required to engage in effective, culturally responsive communication with cases and contacts. Didactic webinars provide (a) fundamental knowledge of technical topics including the epidemiology of COVID-19, comprehensive containment strategies for preventing the spread of COVID-19, and the purpose and practice of case investigation and contact tracing; and (b) a basic understanding of skills important for effective communication with members of diverse communities, including cultural humility, health coaching, and motivational interviewing (MI). Skill development labs allow trainees to actively practice case investigation and contact tracing interviews and build skills in health coaching and MI. Initially (through November 2020), trainees would enroll in either a 16-h CT track or a 20-h CT/CI track, depending on their anticipated job assignment. The core course content was the same, with CI-track learners completing additional didactic and skill development sessions to learn how to conduct case investigation interviews. The CT-only track was discontinued in December 2020 as the state experienced an increased demand for CIs. Additional details about training development and content (7) and evaluation of the impact of the program on trainees' knowledge and self-perceived skills (8) are published elsewhere.

### Purpose of the Evaluation

The purpose of this evaluation was to assess whether trainees felt adequately prepared to perform CI/CT duties after deployment in the field to inform best practices for rapidly scaling and training a workforce to respond to public health emergencies. Given the disparate backgrounds of the new workforce, the primary research question was whether the VTA adequately prepared trainees for deployment regardless of their personal and professional characteristics. Specific aims were to understand trainees' (1) self-perceived proficiency in interviewing skills, and (2) self-perceived job preparedness, and whether these varied by (a) education level, (b) professional background, or (c) volunteer status.

### Population and Setting

From May 18, 2020 to February 26, 2021, 8,141 individuals (29 cohorts of trainees) completed the VTA training. Course completers were defined as trainees who passed a post-training knowledge assessment with a score of 70% or higher within two attempts. The course was offered weekly from May through September to rapidly build workforce capacity, then semi-monthly thereafter as enrollment decreased. Because this was a CDPH-funded initiative, course participation was limited to individuals who were assigned to conduct case investigation and/or contact tracing for a local health department (LHD) or LHD partner organization (e.g., a university or tribal organization), and included primarily county staff (i.e., LHD staff and redirected non-LHD county government staff), and redirected staff from over 100 State departments. Eligibility was verified by the VTA training team and CDPH.

### Data Sources and Measures

A “Real World Feedback” survey was distributed to trainees ~3 weeks following completion of the training. This timeline was intended to be long enough so that trainees were likely to have been deployed and working as CIs/CTs, but not so long to cause significant concern about the probability of recall bias. Initially, an email announcement was sent to all course enrollees ~2.5 weeks after the final day of the course, inviting them to take the optional survey in the course Learning Management System (LMS). Due to concerns that some trainees might have forgotten their LMS log-in credentials and been unable to access the survey, survey administration was moved to the Qualtrics survey platform in September 2020. Another benefit of this transition was the ability for invitations to be targeted only to trainees who received a record of course completion, to ensure feedback was gathered from trainees who fully engaged with the course. Additionally, to improve the likelihood that trainees had begun working as a CI or CT before taking the survey, the timing of the invitation was moved to ~3.5 weeks after course completion. The survey was voluntary, and no incentives were offered for completion. The UCLA Office of the Human Research Protection Program determined that the evaluation project did not meet the definition of human participants research, so did not require institutional review board approval.

The survey was developed by the VTA Monitoring and Evaluation Working Group to reflect the unique content of the training and job responsibilities of COVID-19 CIs and CTs in California. Two measures were used to assess overall preparedness among those who had been deployed to perform CI/CT duties: self-perceived interviewing proficiency and self-perceived job preparedness.

#### Self-Perceived Interviewing Proficiency

Survey respondents were asked to assess their level of comfort and proficiency in the following CI/CT interview areas using a 5-item Likert scale (1 = not proficient, 2 = somewhat proficient, 3 = neutral, 4 = proficient, and 5 = extremely proficient): (a) assessment of symptoms and medical history; (b) assessment of COVID-19 exposure; (c) categorizing a contact's risk; (d) providing guidance on self-isolation and quarantining; (e) referring contacts for testing; and (f) referring contacts for social support services. In addition, a composite interviewing proficiency score was created by summing the six items, with scores possibly ranging from 6 to 30.

#### Self-Perceived Job Preparedness

Survey respondents were asked to indicate their level of agreement with the following six statements about job perception and preparedness using a 5-item Likert scale (1 = strongly disagree; 2 = somewhat disagree; 3 = neither agree nor disagree; 4 = somewhat agree; 5 = strongly agree): (a) I was prepared to do this work; (b) I can do this work well; (c) I am making a difference through this work; (d) I am prepared to use MI skills; (e) I am prepared to use my health coaching skills; (f) I am prepared to handle difficult situations. In addition, a composite job preparedness score was created by summing the six items, ranging from 6 to 30.

Survey data were matched to registration data to obtain age, race and ethnicity, gender, education level, employer, career background, and volunteer status. Education level was classified as less than bachelor's degree (high school, some college, or associate degree); college graduate; or graduate school (some graduate school or graduate degree). Career background was obtained by gathering registrants' professional roles prior to the pandemic and was dichotomized as public health related (disease investigator, public health nurse, public health administrator, other allied health professional) or non-public health related (e.g., librarian, city attorney, etc.). As some state and LHD staff volunteered to perform CI/CT duties, volunteer status was gathered at registration and dichotomized as volunteer (trainees who volunteered to be trained as CIs/CTs) or assigned (trainees who were assigned to be trained through their job); those who reported not knowing their volunteer status were classified as assigned.

### Analysis

We examined descriptive characteristics of the course completers, the sample of survey completers, and the sample of survey completers who had been deployed to perform CI/CT duties at the time of survey completion. Differences between course completers and survey completers were explored using Pearson chi-squared tests. Job preparedness measures were analyzed among survey completers who had been deployed. Self-perceived interviewing proficiency and job preparedness were summarized using means. Bivariate analyses using Wilcoxon rank sum or Kruskal Wallis tests were used to examine differences in individual and composite interviewing proficiency and job preparedness scores by trainee characteristics. Sample sizes vary slightly due to non-response. Analyses were conducted using R version 4.0.3 and Rstudio Version 1.4.1103.

## Results

Among the 8,141 course completers, *n* = 1,522 completed the survey (19% response rate). Of those, *n* = 439 (29%) had been deployed to perform contact tracing duties and represent the primary sample for analyses. Table 1 summarizes the demographic characteristics of course completers, survey completers, and those who had been deployed.

**Table 1:** Demographic characteristics of course participants and study sample.

	**Course completers**		**Survey completers**		**Deployed**	
	**(*n* = 8,141)**		**(*n* = 1,522)**		**(*n* = 439)**	
	***n***	**%**	***n***	**%**	***n***	**%**
**Number of CI/CT interviews conducted at time of survey completion**					**439**	
1–5					164	37.4%
6–10					89	20.3%
11–20					92	21.0%
21+					94	21.4%
**Employer**	**8,123**		**1,519**		**438**	
State of California^a^	3,720	45.8%	908	59.8%**	129	29.5%
County (LHD or Local Government)	3,014	37.1%	446	29.4%	231	52.7%
Other (including Tribal, Clinic, or Community Organizations and Volunteers^b^)	1,389	17.1%	165	10.9%	78	17.8%
**Career Background**	**6,339**		**1,190**		**379**	
Non-public health related	4,474	70.6%	925	77.7%**	255	67.3%
Health or public health related^c^	1,865	29.4%	265	22.3%	124	32.7%
**Highest Education**	**6,878**		**1,306**		**411**	
Less than bachelor's degree^d^	2,168	31.5%	420	32.2%*	101	24.6%
College graduate	2,618	38.1%	442	33.8%	148	36.0%
Graduate school^e^	2,092	30.4%	444	34.0%	162	39.4%
**Volunteer Status**	**6,855**		**1,309**		**407**	
Volunteered to be CI/CT	2,318	33.8%	543	41.5%**	134	32.9%
Assigned or Not Sure	4,537	66.2%	766	58.5%	273	67.1%
**Race**	**6,385**		**1,196**		**384**	
American Indian or Alaska Native	125	2.0%	27	2.3%**	10	2.6%
Asian	1,144	17.9%	181	15.1%	52	13.5%
Black or African American	552	8.6%	102	8.5%	33	8.6%
Native Hawaiian or Pacific Islander	82	1.3%	17	1.4%	5	1.3%
White/Caucasian	2,831	44.3%	629	52.6%	197	51.3%
Two or more	114	1.8%	28	2.3%	6	1.6%
Decline to State	1,537	24.1%	212	17.7%	81	21.1%
**Hispanic**	**6,385**		**1,196**		**384**	
Yes	1580	24.7%	251	21.0%**	96	25.0%
No	4184	65.5%	855	71.5%	245	63.8%
Decline to state	621	9.7%	90	7.5%	43	11.2%
**Gender**	**6,903**		**1,315**		**412**	
Female	4,503	65.2%	894	68.0%**	284	68.9%
Male	1,935	28.0%	367	27.9%	100	24.3%
Transgender, gender queer, or other	36	0.5%	4	0.3%	3	0.7%
Decline to State	429	6.2%	50	3.8%	25	6.1%
**Age**	**6,853**		**1,306**		**406**	
18–24	650	9.5%	51	3.9%**	30	7.4%
25–34	1,396	20.4%	153	11.7%	66	16.3%
35–44	1,216	17.7%	208	15.9%	65	16.0%
45–54	1,331	19.4%	346	26.5%	87	21.4%
55–64	1,213	17.7%	329	25.2%	83	20.4%
65 plus	481	7.0%	143	10.9%	43	10.6%
Decline to State	566	8.3%	76	5.8%	32	7.9%

CI, case investigation; CT, contact tracing; LHD, local health department. Notes: Pearson chi-square tests examined differences in demographic characteristics between course completers and survey completers; ^*^significant at p < 0.01, ^**^significant at p < 0.001; ^a^Includes employees contracted to work for State departments by organizations such as Heluna Health and CDC Foundation; ^b^A small number of LHD's permitted community volunteers to participate in CT/CI efforts; ^c^Health or public health related professional roles include disease investigator, public health nurse, public health administrator, or other allied health professional; ^d^Less than bachelor's degree includes those with high school, some college, or 2-year degree; ^e^Graduate school includes those with some graduate school or graduate degree.

Survey completers were primarily redirected State of California staff (60%), from non-public health-related careers (78%), and assigned to work in the COVID-19 response (59%). Approximately one-third of survey respondents had completed less than a bachelor's degree (32%). Just over half of the respondents were White (53%) and two-thirds were female (68%). Nearly two-thirds of survey respondents were age 45 years and older (63%). Compared to the full sample of course completers, survey respondents were more likely to be State of California staff (60% vs. 46%, *p* < 0.001), from non-public health-related fields (78% vs. 71%, *p* < 0.001), and volunteers (42% vs. 34%, *p* < 0.001).

Table 2 summarizes self-perceived interviewing proficiency and self-perceived job preparedness. Mean scores for most interviewing skills were at least 4, corresponding to “proficient” on the Likert scale. Trainees were most confident in their ability to provide guidance on self-isolation and quarantining (proficiency score = 4.2). The only item with a mean score below 4 related to self-perceived proficiency referring contacts for social support services (3.7). Similarly, mean scores for most job preparedness items were at least 4. Trainees had strong convictions in their ability to do contact tracing work well (preparedness score = 4.3) and their ability to make a difference through their work (preparedness score = 4.4). Trainees felt least prepared to handle difficult situations, with a mean score of 3.8.

**Table 2:** Self-perceived interviewing proficiency (*n* = 409) and job preparedness (*n* = 412).

**Preparedness Measures**	**Mean score**
**Interviewing Proficiency** (*n =* 409)	**Proficiency score** ^**a**^
**Individual Interview Skill Items**	
Guidance on self-isolation and quarantining	4.2
Assessment of symptoms and medical history	4.1
Assessment of COVID-19 exposure	4.1
Referring contacts for testing	4.0
Categorizing contact's risk	4.0
Referring contacts for social support services	3.7
Composite interviewing proficiency score^b^	24.0
**Job Preparedness** (*n =* 412)	**Preparedness score** ^**c**^
**Individual Job Preparedness Items**	
I am making a difference through this work	4.4
I can do this work well	4.3
I am prepared to use motivational interviewing skills	4.2
I am prepared to use my health coaching skills	4.0
I was prepared to do this work	4.0
I am prepared to handle difficult situations	3.8
Composite job preparedness score^d^	24.6

a *Respondents rated proficiency on a scale from 1 (not proficient) to 5 (extremely proficient)*;

b *Composite proficiency score is the sum of the six interview skill proficiency scores and can range from 6 to 30*;

c *Respondents rated preparedness on a scale from 1 (strongly disagree) to 5 (strongly agree)*;

d *Composite preparedness score is the sum of the six job preparedness item scores and can range from 6 to 30*.

**Figure 1:**
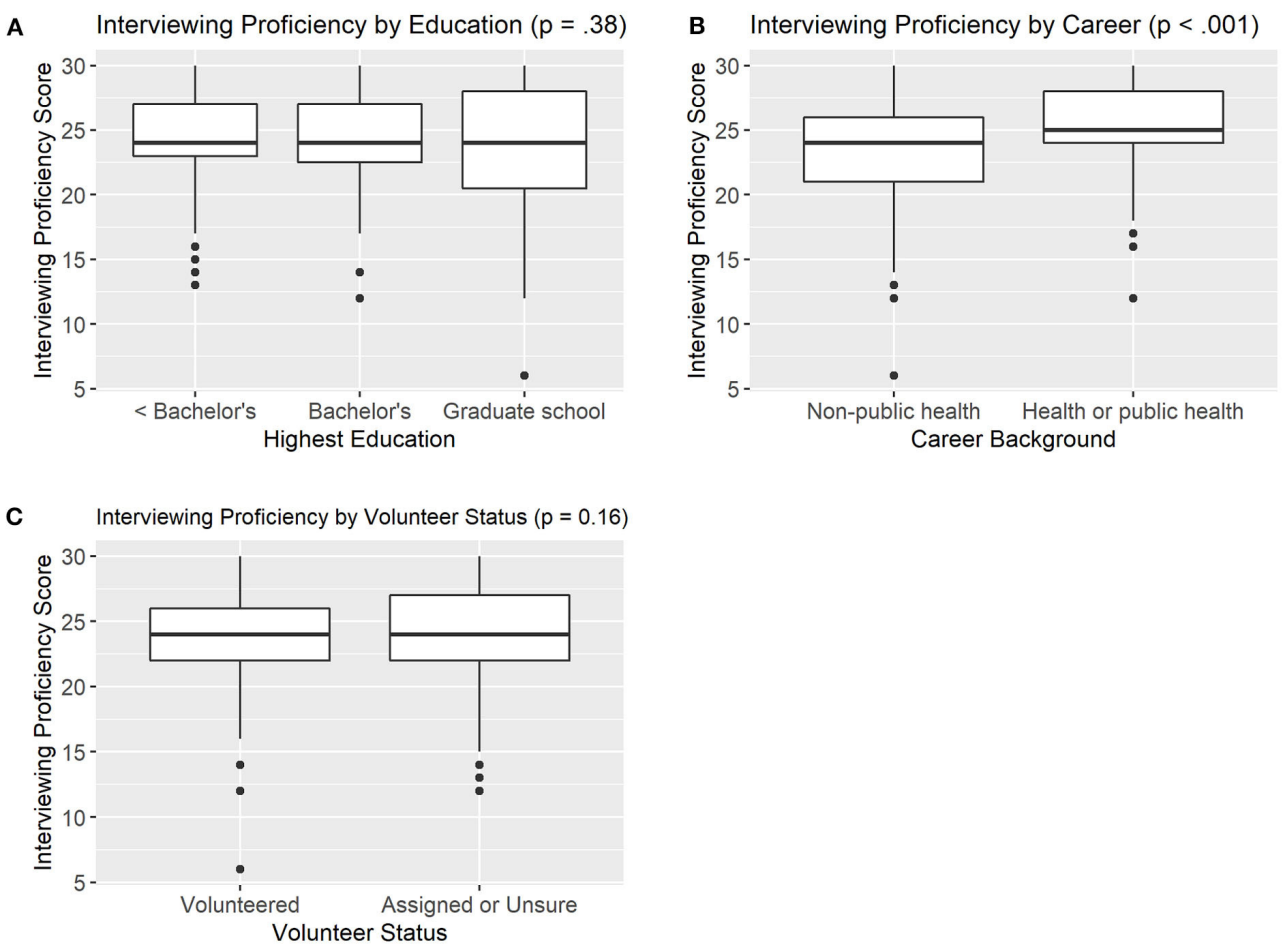
Differences in Self-Perceived Interviewing Proficiency by Education Level (**A**, *n* = 383), Career Background (**B**, *n* = 353), and Volunteer Status (**C**, *n* = 380).

Figure 1 summarizes differences in self-perceived interviewing proficiency by education (A), career background (B) and volunteer status (C).

There were no significant differences in composite interviewing proficiency scores between trainees with less than bachelor's degree (24.2), college (24.2), or graduate school (23.5) education (*p* = 0.38). Similarly, there were no differences in self-perceived interviewing proficiency between those who volunteered to be trained as CIs/CTs (23.5) and those who were assigned by their employer (24.1) (*p* = 0.16). However, there were significant differences in interviewing proficiency by career background. Trainees with public health career backgrounds had significantly higher self-perceived proficiency in interviewing skills (25.1) compared to those with non-public health backgrounds (23.3) (*p* < 0.001). In examining the six interview skills individually, trainees with public health backgrounds had higher self-perceived proficiency on all six items compared to those with non-public health backgrounds (*p* < 0.05, data not shown).

**Figure 2:**
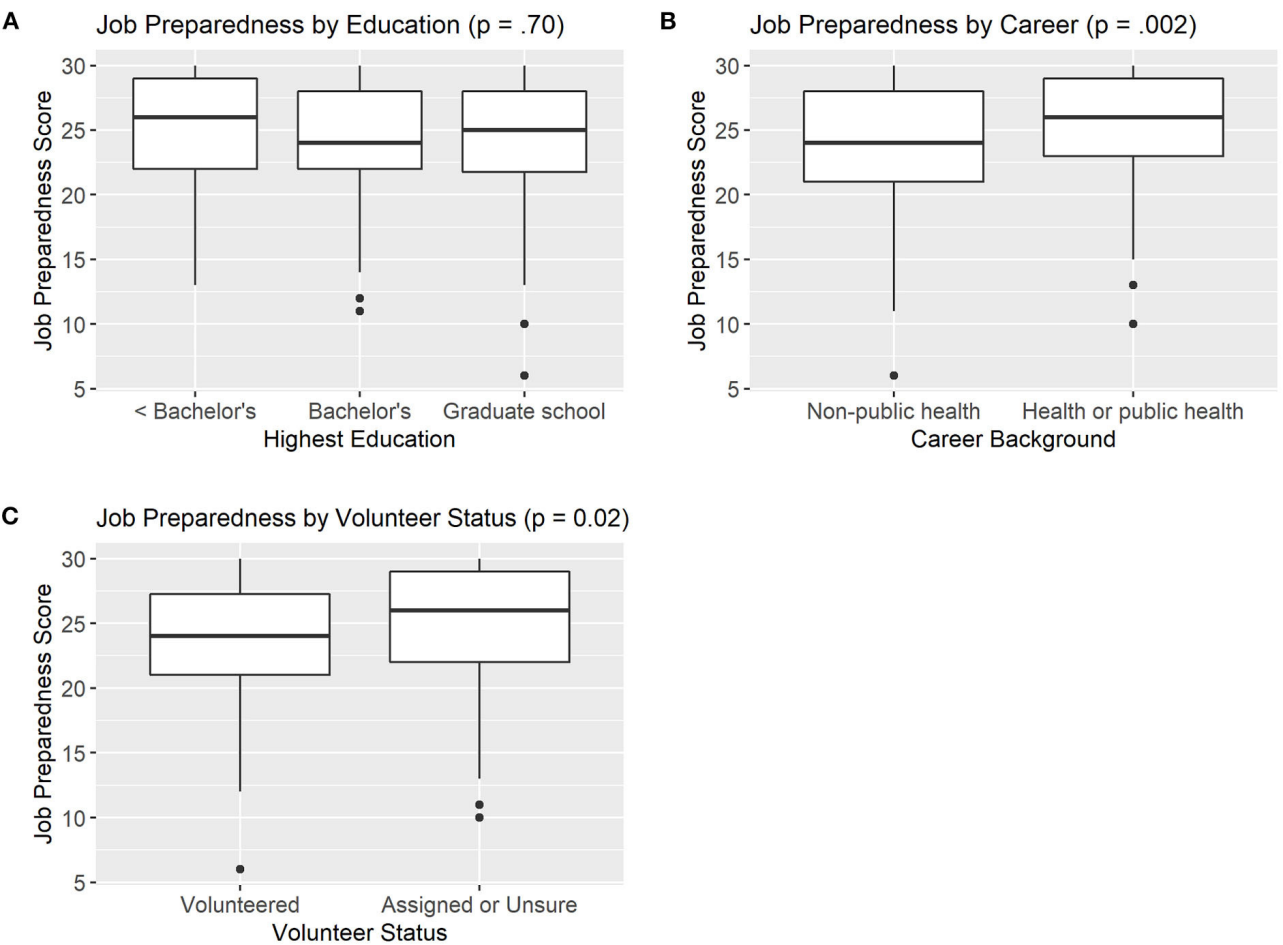
Differences in Self-Perceived Job Preparedness by Education Level (**A**, *n* = 386), Career Background (**B**, *n* = 356), and Volunteer Status (**C**, *n* = 383).

Figure 2 summarizes differences in self-perceived job preparedness by education **(A)**, career background **(B)**, and volunteer status **(C)**.

There were no differences in composite job preparedness scores between trainees with less than bachelor's degree (24.9), college (24.5), or graduate school (24.4) education (*p* = 0.70). However, there were differences in job preparedness by career background and volunteer status. Trainees with public health backgrounds reported greater preparedness to perform contact tracing job duties (25.7) than those with non-public health backgrounds (24.0) (*p* < 0.01). Examination of the individual job preparedness items revealed there was no significant difference in self-perceived preparedness to use MI skills between those with public health and non-public health backgrounds (*p* = 0.06, data not shown). However, those with public health backgrounds had significantly higher self-perceived preparedness to handle difficult situations, use health coaching skills, do the work and do it well, and make a difference (*p* < 0.05, data not shown).

Volunteers had significantly lower job-preparedness scores (23.8) compared to those who were assigned (24.9) (*p* = 0.02). In examining the individual job preparedness items, there were three key indicators driving this significant difference. There was no difference between volunteers and non-volunteers in their perceived ability to use health coaching skills in their work, or their perception that they can do the work well and are making a difference through the work (*p* > 0.05, data not shown). However, volunteers scored significantly lower on preparedness to do the work (*p* < 0.01), to use MI skills (*p* = 0.04), and to handle difficult situations (*p* < 0.01) compared to CI/CT staff who did not volunteer (data not shown).

## Discussion and Lessons Learned

Federal, state, and local health agencies had to act swiftly to train a competent workforce of case investigators and contact tracers to effectively fight the spread of COVID-19 in the U.S. Recruitment and training efforts varied widely (9), and few studies to date have described the workforce or evaluated the effectiveness of these training programs. This evaluation suggests that individuals without health backgrounds and those without bachelor's or advanced degrees can be rapidly trained and deployed to conduct disease investigation duties, and provides support for expanding and diversifying the public health workforce pipeline.

One key finding was that there was no difference in preparedness by educational background. Approximately one-third of all VTA trainees and one-quarter of survey respondents who had been deployed as CIs/CTs had less than a bachelor's degree. However, they were similarly confident in their interviewing proficiency and their ability to perform CI/CT duties as well as more diverse as those with college or graduate degrees. This has important implications for strategic development of the public health workforce, one of which is the value of non-4-year college degree training programs (i.e., accelerated “pipeline” programs) to train entry-level public health workers. A recent survey of staff in State Health Agencies found that 75% of the workforce had college degrees (10), which reflects a significant barrier to entry. Accelerated pipeline programs not only increase the speed at which the public health workforce can be trained, but also can increase the racial and ethnic diversity of the workforce to reflect and represent the diverse populations served by public health departments, better equipping them to address health disparities (11, 12). In addition, these findings provide a compelling case for health departments to leverage current federal funding opportunities—such as the American Rescue Plan—to invest in community health workers who may not have advanced education but who have community knowledge and shared lived experience (13).

While there were no differences in preparedness by education, there were statistically significant differences by career background, with trainees who entered the workforce from public health or allied health fields feeling more prepared to perform CI/CT work. There are several potential factors that may contribute to these differences. First, health professionals may work in settings where they perform job duties similar to CIs/CTs, including assessing medical histories and providing referrals to social support services. In addition, there are several “soft skills” that are critical to the success of disease investigation work which may not be core functions of some non-health careers, including establishing rapport and building trust. The importance of these skills are demonstrated by a survey conducted by the California Health Care Foundation which found that about 4 in 10 Californians were unwilling or potentially unwilling to share personal information about their health, movements, and contacts with public health officials (14). However, though differences were statistically significant, the practical significance was small (<2-point differences on 30-point composite scores), so the implications for public health practice and outcomes may be minimal. Furthermore, certain non-health fields do have considerable transferrable skills that make them particularly adept at performing disease investigation duties. As an example, anecdotal evidence from the COVID-19 pandemic response suggests that redirected staff such as librarians and tax assessors are skilled in contact tracing work because they are accustomed to having conversations about sensitive and difficult topics (15). Thus, for future public health emergencies, these findings suggest that when the non-health workforce is critical to expanding public health capacity, there may be benefit to targeted recruitment of fields with key transferrable skills relevant to the emergency response duties.

This evaluation revealed two areas in which deployed trainees were slightly less confident in their abilities. The first was handling difficult situations, which was the only job preparedness item with a score that trended toward neutral, or uncertainty of whether they were prepared. While the VTA skill development labs provided opportunities for trainees to practice interviews, additional sessions with more complex scenarios may be warranted to improve self-efficacy before deployment. The second area with comparatively lower self-perceived proficiency was the ability to refer contacts for social support services. While the VTA provided training on how to elicit information to determine whether cases may need social support services, the actual availability of such services and the process by which to refer could vary widely across jurisdictions and is therefore contextual knowledge better suited for local training post-deployment. In addition, this may reflect the reality that the capacity of social safety net programs were often stretched during the COVID-19 pandemic due to increased demand, as well as the need for programs to adapt their service provision models to protect their workers and clients (16).

One unexpected finding was that individuals who reported that they volunteered to join the contact tracing workforce had lower self-perceived preparedness compared to those who were assigned to the role. Our a priori hypothesis was that volunteers may have greater enthusiasm about their role and more active engagement in the training program, which may translate into greater preparedness to perform contact tracing work. In contrast, we actually found that volunteers had lower self-perceived job preparedness than non-volunteers, driven by significantly lower perceptions of their overall preparedness to do CI/CT work, to apply MI skills, and to handle difficult situations. One hypothesis, derived from theories of cognitive bias (17), is that volunteers may have underestimated their competence because they placed greater importance on the work. Because individuals who volunteered are likely to be highly motivated and committed to performing the job well, they may have higher expectations for themselves than those who were assigned. Another important consideration is that redirected state department staff, who were primarily assigned, had additional opportunities for support provided by CDPH and the VTA, including access to a Mentorship Team of experienced CIs, as well as virtual Communities of Practice to continue to build knowledge and skills.

### Limitations

One limitation of this study is potential non-response bias due to the low response rate. It is possible that those who responded to the survey had different perceptions about their preparedness compared to those who did not. One challenge was the inability to incentivize survey completion, as employees of the State of California are not permitted to receive gift cards or other forms of incentives. In addition, there were limitations with both methods of survey administration. While we moved to Qualtrics to limit barriers to accessing the survey, in using Qualtrics to administer subsequent program evaluation surveys there were reports that some LHD e-mail servers block or quarantine e-mails sent from the Qualtrics platform, suggesting that some trainees may not have received the survey invitation. Finally, California experienced a significant surge in COVID-19 cases between November 2020–February 2021, with CTs in some counties receiving up to 4,000 call assignments per day (18, 19). Increased caseloads and subsequent burnout likely reduced their capacity to respond to the survey in a timely manner.

Another limitation is the potential for recall bias, namely that as length of CI/CT deployment increases, trainees' knowledge and skills improve and they have difficulty recalling their preparedness at initial deployment. However, we attempted to limit the potential for recall bias by administering the survey within 3 weeks post course completion. Finally, this evaluation reflects self-perceived preparedness, not competency, so more research is necessary to assess how the VTA training impacted California's response to the COVID-19 pandemic. Additional priorities for future research include understanding how demographic characteristics of trainees, including race/ethnicity and languages spoken, impacted the perceived preparedness and performance of California's CI/CT workforce, particularly given the disparate impact of COVID-19 on communities of color (20). Despite potential limitations, this evaluation provides valuable insight into trainees' perception of their preparedness to work as CIs/CTs following a weeklong virtual training program.

## Conclusion

The COVID-19 pandemic emphasized the critical need to reimagine who delivers essential public health services in the U.S. In order to engage effectively with community members, a contact tracing workforce needs to be empathetic, language concordant, and culturally responsive (21, 22). To achieve this goal, there must be focused efforts to diversify the public health workforce, including recruitment of professionals from the communities they will serve and from backgrounds underrepresented in the field. This evaluation provides evidence that individuals outside of the traditional public health pipeline, including those without bachelor's or advanced degrees and those without health or public health experience, can be rapidly trained and deployed and feel confident and prepared to perform disease investigation duties, lending critical surge capacity at an essential time.

## Data Availability Statement

The raw data supporting the conclusions of this article will be made available by the authors, without undue reservation.

## Author Contributions

KW, MR, AD, and MP contributed to the conception and design of the project. MW, MF, OG, and SS contributed to the conceptualization of the paper and review and synthesis of the literature. OG and SS contributed to acquisition of data. MW, OG, KT, and SS contributed to analysis and/or interpretation of the data. MW and MF wrote the first draft of the manuscript. All authors contributed to critical review and revision of the manuscript.

## Funding

This work was supported by the California Department of Public Health [agreement number 19-11102].

## Author Disclaimer

The contents may not necessarily reflect the official views or policies of the State of California.

## Conflict of Interest

The authors declare that the research was conducted in the absence of any commercial or financial relationships that could be construed as a potential conflict of interest.

## Publisher's Note

All claims expressed in this article are solely those of the authors and do not necessarily represent those of their affiliated organizations, or those of the publisher, the editors and the reviewers. Any product that may be evaluated in this article, or claim that may be made by its manufacturer, is not guaranteed or endorsed by the publisher.
